# Sarcopenic obesity is part of obesity paradox in dementia development: evidence from a population-based cohort study

**DOI:** 10.1186/s12916-024-03357-4

**Published:** 2024-03-22

**Authors:** Junhan Zhang, Xiaona Na, Zhihui Li, John S. Ji, Guowei Li, Haibing Yang, Yucheng Yang, Yuefeng Tan, Jian Zhang, Menglu Xi, Donghan Su, Huatang Zeng, Liqun Wu, Ai Zhao

**Affiliations:** 1 Vanke School of Public Health, Tsinghua University, Beijing, China; 2 Institute for Healthy China, Tsinghua University, Beijing, China; 3 Center for Clinical Epidemiology and Methodology, Guangdong Second Provincial General Hospital, Guangzhou, China; 4 School of Public Health, Peking University, Beijing, China; 5 Shenzhen Health Development Research and Data Management Center, Shenzhen, Guangdong China

**Keywords:** Sarcopenic obesity, Obesity, Sarcopenia, Dementia, Onset, Brain structure

## Abstract

**Background:**

Sarcopenic obesity, a clinical and functional condition characterized by the coexistence of obesity and sarcopenia, has not been investigated in relation to dementia risk and its onset.

**Methods:**

We included 208,867 participants from UK biobank, who aged 60 to 69 years at baseline. Dementia diagnoses were identified using hospital records and death register data. Hazard ratios (HRs) and 95% confidence intervals (CIs) were estimated using Cox proportional hazards models to evaluate the associations of obesity, sarcopenia, and sarcopenic obesity with dementia risk, stratified by sex. Stratified analyses were performed across dementia-related polygenic risk score (PRS). Restricted mean survival time models were established to estimate the difference and 95%CIs of dementia onset across different status. Additionally, linear regression models were employed to estimate associations of different status with brain imaging parameters. The mediation effects of chronic diseases were also examined.

**Results:**

Obese women with high PRS had a decreased risk (HR = 0.855 [0.761–0.961]), but obese men with low PRS had an increased risk (HR = 1.223 [1.045–1.431]). Additionally, sarcopenia was associated with elevated dementia risk (HR_women_ = 1.323 [1.064–1.644]; HR_men_ = 2.144 [1.753–2.621]) in those with low PRS. Among those with high PRS, however, the association was only significant in early-life (HR_women_ = 1.679 [1.355–2.081]; HR_men_ = 2.069 [1.656–2.585]). Of note, sarcopenic obesity was associated with higher dementia risk (HR_women_ = 1.424 [1.227–1.653]; HR_men_ = 1.989 [1.702–2.323]), and results remained similar stratified by PRS. Considering dementia onset, obesity was associated with dementia by 1.114 years delayed in women, however, 0.170 years advanced in men. Sarcopenia (women: 0.080 years; men: 0.192 years) and sarcopenic obesity (women: 0.109 years; men: 0.511 years) respectively advanced dementia onset. Obesity, sarcopenia, and sarcopenic obesity were respectively related to alterations in different brain regions. Association between sarcopenic obesity and dementia was mediated by chronic diseases.

**Conclusions:**

Sarcopenic obesity and sarcopenia were respectively associated with increased dementia risk and advanced dementia onset to vary degree. The role of obesity in dementia may differ by sex and genetic background.

**Supplementary Information:**

The online version contains supplementary material available at 10.1186/s12916-024-03357-4.

## Background

Dementia is a collective term encompassing a range of brain disorders that diminish cognitive function, such as memory, thinking, and emotion. Globally, it is projected that the number of dementia cases is about to shoot up from 57 million in 2019 to 152 million by 2050, the majority of which happen among the greying population [1]. Dementia has posed a substantial societal and economic burden. From 2000 to 2019, disability-adjusted life years of dementia has increased 122% [2]. In 2019, the global costs of dementia were estimated at around US$1.3 trillion, and this figure is projected to exceed US$2.8 trillion by 2030 [3]. Considering the tremendous consequences of dementia, there is a growing focus on understanding and addressing its onset [4]. It is reckoned that the dementia onset will remain relatively stable until 2050, but individuals may live longer with the condition due to the increasing life expectancy [1, 5]. It is predicted that delaying onset of dementia for 2 years there would be nearly 2 million fewer cases in 2050 [6].

The causes of dementia have not been fully understood. Numerous studies have explored various factors that may affect the risk of dementia, including aging, obesity, sarcopenia, chronic diseases, and genetics. However, there is a lack of consensus regarding the relationship between obesity and dementia. While some research has suggested that late-life obesity may serve as an independent risk factor for dementia, other studies have reported a reduced risk of dementia in obese individuals, a phenomenon known as the “obesity paradox” [7]. Additionally, sarcopenia has also been proposed to be a potential risk factor to dementia [8]. In recent years, increasing negative impacts from sarcopenic obesity, a clinical and functional condition characterized by the coexistence of obesity and sarcopenia, have been reported [9], indicating that there may exist amplified health risks of sarcopenic obesity than singular obesity or sarcopenia.

Although there were a few observational studies suggested a potential association between sarcopenic obesity and a higher risk of cognitive impairment [10, 11], the most significant characteristic of dementia, limited research has reported the association between sarcopenic obesity and dementia risk. In addition, to our knowledge, there are no studies working on the association between sarcopenic obesity and the onset of dementia. Considering the rising prevalence of sarcopenic obesity [12], whether it will augment the risk or/and advance the onset of dementia is worth to be investigated. Moreover, to further understand the underlying mechanisms, there is a great need to investigate and compare the effect from obesity, sarcopenia, and sarcopenic obesity on brain structure. Previous studies have reported the associations of obesity or sarcopenia with brain structure, but not sarcopenic obesity, and there is no unified conclusion [13, 14]. Additionally, since sarcopenic obesity and dementia are both related to cardiovascular disease (CVD) [15, 16], cerebrovascular disease (CeVD) [17, 18], diabetes [19, 20], and depression [21, 22] whether they act as mediators should be examined. Furthermore, genetic background, such as apolipoprotein E epsilon 4 (APOE4) [23], has been demonstrated to be the important risk factor of dementia; whether there exists the moderation effect remains to be explored.

To address these gaps, prospective data from UK Biobank was utilized in this study to explore: (1) the associations of obesity, sarcopenia, and sarcopenic obesity with the incidence and onset of dementia, and brain structure; (2) the roles of genetic background, CVD, CeVD, diabetes, and depression.

## Methods

### Study design and participants

This prospective population-based cohort study was based on UK Biobank, which recruited over half million participants aged 40–69 years to attend baseline assessment between 2006 and 2010, at 22 assessment centers across England, Scotland, and Wales. More detailed information of UK Biobank is available in previous studies [24, 25].

**Fig. 1:**
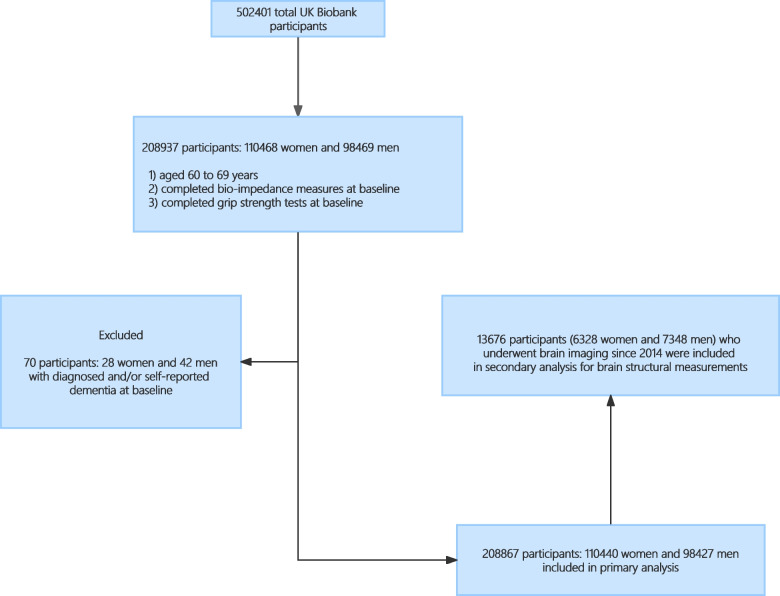
Flowchart of participant enrolment

In current study design, 208,937 participants (110,468 women and 98,469 men) who aged 60 to 69 years and completed both bio-impedance measures and grip strength tests at baseline were eligible for inclusion. Participants with diagnosed and/or self-reported dementia at baseline, amounting to 70 individuals (28 women and 42 men), were excluded from the analysis. In total, our primary analysis involved 208,867 participants (110,440 women and 98,427 men), among whom 13,676 participants (6328 women and 7348 men) have undergone brain image assessments since 2014 and were included in the secondary analysis for exploring the associations of obesity, sarcopenia, and sarcopenic obesity with brain structural parameters. The flow path of inclusion and exclusion was presented in Fig. 1.

### Assessment of obesity

BMI was estimated with baseline height, which was measured to the nearest 0.1 centimeter (cm) using a Seca 202 height measure, and baseline weight, which was measured to the nearest 0.1 kilogram (kg) using a Tanita BC418MA body composition analyzer. Obesity was defined as BMI ≥ 30 kg/m^2^, in accordance with the WHO criteria [26]. In present study, participants who met the criteria of obesity but not sarcopenia at baseline were categorized into obesity group.

### Assessment of sarcopenia

Sarcopenia was defined as the combination of low muscle strength and low muscle quantity, according to the European Working Group on Sarcopenia in Older People 2 (EWGSOP2) [27]. Grip strength (the mean values of the right and left hand), measured with a Jamar J00105 hydraulic hand dynamometer at baseline, was used to estimate the muscle strength. The cut-off points used to define low grip strength were < 27 kg in men and < 16 kg in women [27]. Skeletal muscle mass index (SMI) was generated using bioelectrical impedance analysis (BIA). Specifically, SMI was calculated from appendicular skeletal muscle mass (ASM) divided by height squared. ASM was derived from Janssen equation [28], utilizing the resistance value obtained through Tanita BC418MA body composition analyzer. Low muscle quantity was defined as SMI < 7.0 kg/m^2^ for men and < 5.5 kg/m^2^ for women [27]. Participants who met the criteria of sarcopenia but not obesity at baseline were categorized into sarcopenia group.

### Identification of sarcopenic obesity

In present study, participants who met both criteria of obesity and sarcopenia at baseline were categorized into sarcopenic obesity group. In addition, a “normal” reference group consisted of those without obesity, sarcopenia, or sarcopenic obesity.

### Assessment of dementia

The primary outcome of the study was the incident dementia occurred during the follow-up, which was ascertained based on linked hospital-admission data (Hospital Episode Statistics for England (HES), Scottish Morbidity Record (SMR), and the Patient Episode Database for Wales (PEDW)) and death register data (National Health Service (NHS) Digital (England and Wales), NHS Central Register, and National Records (Scotland)). Additionally, hospital-admission data were available until 30 November 2022, and death register data were available up to 30 November 2022 for England, 31 July 2021 for Scotland, and 28 February 2018 for Wales. Therefore, the follow-up period was calculated from the baseline to the first record of incident dementia, death, loss to follow-up, or the last follow-up date, whichever occurred first. Incident dementia was defined as a hospital admission or death with the following International Classification of Diseases 10th revision (ICD-10) or ICD-9 codes: ICD-10 codes A81.0, F00-F03, G30, G31.0, G31.1 and G31.8, and ICD-9 codes 290.2-290.4, 291.2, 294.1, 331.0-331.2, and 331.5.

### Assessment of brain structure

The secondary outcomes of this study were the volume (mm^3^) of the total brain, white matter, and grey matter generated from T1 structural brain magnetic resonance imaging (MRI) and white matter hyperintensities (WMHs) derived from T2-weighted brain MRI. The volume of the total brain, white matter, and grey matter were normalized for the external surface of the skull from the T1 MRI. Both left and right of normalized brain volume were summed. The volume of WMHs, with a skewed distribution, were log-transformed. All brain imaging measurements were conducted using a standard Siemens Skyra 3T running VD13A SP4, with a standard Siemens 32-channel RF receive head coil since 2014.

### Assessment of mediators

A hospital admission or death, with the same data source as incident dementia, based on ICD-10 codes were used to diagnose CVD (I20-I22, I46, and I50), CeVD (I60-I70), diabetes (E10-E14), and depression (F32-F33), which included baseline cases and the ones developed during follow-up.

### Assessment of covariates

The baseline questionnaire was used to assess the following potential confounders: (1) sociodemographic factors: age, sex, ethnicity, education qualifications, Townsend Deprivation Index (TDI); (2) lifestyles: physical activity, smoking status, alcohol status, dietary intake (vegetables, fruits, red meat, process meat, oily fish, and dairy); (3) genetic factor: polygenic risk score (PRS) for Alzheimer’s disease (AD). Ethnicity was categorized as White, Asian or Asian British, Black or Black British, and others. The TDI is an indicator of socioeconomic status, and positive TDI values indicate relative privation. Physical activity was categorized as low, moderate, and high level, according to the guidelines of the International Physical Activity Questionnaire (IPAQ) [29]. Smoking or alcohol status were categorized as current, former, or never user. Frequency of habitual intake of foods was investigated at baseline. Vegetable consumption was measured as average number of heaped tablespoons of vegetables consumed per day. Fruits consumption was categorized as 0–1, 2–3, and ≥ 3 pieces per day (counted one apple, one banana, 10 grapes, etc., as one piece). Meat consumption (pork, beef, lamb, process meat, and oily fish) was categorized as never, less than once a week, once a week, and more than twice a week. Coffee intake was measured as average number of cups of coffee consumed per day. Dairy consumption was categorized as yes or no. The standard PRS for AD was calculated by meta-analyzing two external genome wide association study sources, which could be used for assessing the AD genetic susceptibility, and a higher PRS indicated more risk for AD [30]. In current study, PRS was categorized as low and high level by its median.

### Statistical analysis

Baseline characteristics of the participants were summarized across four groups (normal, obesity, sarcopenia, and sarcopenic obesity) as number and percentage for categorical variables and mean and standard deviation for continuous variables. It is assumed that data were missing at random, and we used multiple imputation chain-equation (MICE) to impute missing covariate values [31]. We generated 5 datasets with 100th iteration by random forest method.

Since progression of obesity and sarcopenic obesity differs between women and men, all analyses were stratified by sex [32, 33]. Kaplan-Meier analyses were performed to calculate cumulative incidence probabilities of all-cause dementia. Time-dependent Cox proportional hazards models were used to estimate the hazard ratios (HRs) and 95% confidence intervals (CIs) for the associations of obesity or sarcopenic obesity with risk of all-cause dementia. On the other hand, segmented Cox regression models were used to evaluate the HRs (95%CIs) between sarcopenia and all-cause dementia risk, with a follow-up time equal to 8 years as the threshold, according to principle of minimizing -2 Log Likelihood value. Schoenfeld residuals test was conducted to test proportional hazard assumption. We calculated the attributable risk proportion (AR%): $\textrm{AR}\%=\frac{\textrm{HR}-1}{\textrm{HR}}\times 100\%$. Three models were further adjusted. Unadjusted models were adjusted by no covariates; age-adjusted models were adjusted by age; multivariable models were adjusted by sociodemographic factors (age, TDI, ethnicity, education), lifestyle (physical activity, smoking status, alcohol status), and dietary intake (vegetables, fruits, red meat, processed meat, oily fish, coffee and dairy). Further stratified analyses were performed to estimate potential modification effect according to PRS levels. Furthermore, we did several sensitivity analyses to test the robustness of our findings. Firstly, we excluded participants who had a dementia event during the first year of follow-up to minimize the influence of reverse causation. We also assessed the influence of death as a competing risk for incident dementia via competing risk analyses [34]. Restricted mean survival time (RMST) models were established to estimate the difference and 95%CIs of dementia onset among four groups [35]. We employed linear regression models to estimate the *β* coefficients and 95% CIs for associations of different groups with brain structure in participants who had undergone image assessments. Additionally, we examined the mediation effect of CVD, CeVD, diabetes, and depression in the association between sarcopenic obesity and dementia risk with logistic regression [36].

We generated a directed acyclic graph (DAG), presented in Additional file 1: Figure S1, to depict the conceptual framework of our analyses.

All statistical analyses were done in R (version 4.2.2). We used the following packages: “mice” for missing data imputation, “survival” and “survminer” for Kaplan-Meier curves and Cox models, “survRM2” for restricted mean time survival models, “mediation” for mediations analyses, and “cmprsk” for competing risk models. The two tailed significance level was set at *P* < 0.05.

## Results

### Baseline characteristics

Baseline characteristics were showed in Table 1. Overall, 22241 (20.1%), 11830 (10.7%), and 4837 (4.4%) women were identified with obesity, sarcopenia, and sarcopenic obesity, respectively; while 22328 (22.7%), 6475 (6.6%), and 2676 (2.7%) men were categorized with obesity, sarcopenia, and sarcopenic obesity, respectively. In comparison to participants in the normal group, both women and men with sarcopenia or sarcopenic obesity were more likely to be old and have a high PRS level, while individuals with obesity were more possible to be young and have a low PRS level. Participants with obesity were likely to have a degree, have no CeVD, and be a former smoker and a current drinker. Participants with sarcopenia were likely to have diabetes and CVD and never smoke. Participants with sarcopenic obesity were likely to have no education degree, low physical activity level, and large TDI.

**Table 1:** Baseline characteristics for women and men stratified by different status (obesity, sarcopenia, and sarcopenic obesity)

	Women					Men				
Characteristic	Normal	Obesity	Sarcopenia	Sarcopenic obesity	*P*	Normal	Obesity	Sarcopenia	Sarcopenic obesity	*P*
	(*n* = 71532)	(*n* = 22241)	(*n* = 11830)	(*n* = 4837)		(*n* = 66948)	(*n* = 22328)	(*n* = 6475)	(*n* = 2676)	
Skeletal muscle mass index, mean (SD), kg/m^2^	3.37 (0.30)	3.81 (0.38)	3.29 (0.31)	3.78 (0.41)	< 0.001	5.27 (0.37)	5.77 (0.42)	5.19 (0.40)	5.73 (0.43)	< 0.001
Grip strength, mean (SD), kg	22.98 (4.37)	22.93 (4.52)	12.25 (2.98)	12.01 (3.09)	< 0.001	38.65 (6.65)	38.72 (7.02)	22.17 (4.07)	21.75 (4.42)	< 0.001
Age, mean (SD), years	63.89 (2.78)	63.86 (2.79)	64.45 (2.83)	64.34 (2.82)	< 0.001	64.14 (2.80)	64.04 (2.78)	64.78 (2.85)	64.66 (2.81)	< 0.001
Ethnicity, *n* (%)					< 0.001					< 0.001
White	69990 (97.8)	21440 (96.4)	11276 (95.3)	4570 (94.5)		65258 (97.5)	21862 (97.9)	5886 (90.9)	2529 (94.5)	
Asian or Asian British	515 (0.7)	171 (0.8)	335 (2.8)	128 (2.6)		782 (1.2)	150 (0.7)	393 (6.1)	82 (3.1)	
Black or Black British	366 (0.5)	405 (1.8)	52 (0.4)	77 (1.6)		416 (0.6)	148 (0.7)	75 (1.2)	33 (1.2)	
Other	661 (0.9)	225 (1.0)	167 (1.4)	62 (1.3)		492 (0.7)	168 (0.8)	121 (1.9)	32 (1.2)	
Education, *n* (%)					< 0.001					< 0.001
No degree	17646 (24.7)	7198 (32.4)	3902 (33.0)	2042 (42.2)		15185 (22.7)	7031 (31.5)	2078 (32.1)	1125 (42.0)	
Degree	53886 (75.3)	15043 (67.6)	7928 (67.0)	2795 (57.8)		51763 (77.3)	15297 (68.5)	4397 (67.9)	1551 (58.0)	
Townsend deprivation index, mean (SD)	− 1.81 (2.78)	− 1.14 (3.11)	− 1.37 (2.98)	− 0.49 (3.28)	< 0.001	− 1.79 (2.86)	− 1.28 (3.09)	− 0.71 (3.35)	− 0.26 (3.41)	< 0.001
Physical activity level, *n* (%)					< 0.001					< 0.001
Low	9244 (12.9)	4802 (21.6)	2039 (17.2)	1394 (28.8)		9683 (14.5)	5175 (23.2)	1364 (21.1)	796 (29.7)	
Moderate	31534 (44.1)	10013 (45.0)	5235 (44.3)	2085 (43.1)		26866 (40.1)	8863 (39.7)	2731 (42.2)	1060 (39.6)	
High	30754 (43.0)	7426 (33.4)	4556 (38.5)	1358 (28.1)		30399 (45.4)	8290 (37.1)	2380 (36.8)	820 (30.6)	
Smoking status, *n* (%)					< 0.001					< 0.001
Never	41364 (57.8)	12174 (54.7)	6981 (59.0)	2721 (56.3)		29872 (44.6)	7596 (34.0)	2854 (44.1)	931 (34.8)	
Former	25193 (35.2)	8770 (39.4)	3970 (33.6)	1807 (37.4)		30360 (45.3)	12773 (57.2)	2789 (43.1)	1499 (56.0)	
Current	4975 (7.0)	1297 (5.8)	879 (7.4)	309 (6.4)		6716 (10.0)	1959 (8.8)	832 (12.8)	246 (9.2)	
Drinking status, *n* (%)					< 0.001					< 0.001
Never	3942 (5.5)	1810 (8.1)	1056 (8.9)	611 (12.6)		1517 (2.3)	539 (2.4)	317 (4.9)	119 (4.4)	
Former	2306 (3.2)	1080 (4.9)	589 (5.0)	380 (7.9)		1986 (3.0)	848 (3.8)	392 (6.1)	189 (7.1)	
Current	65284 (91.3)	19351 (87.0)	10185 (86.1)	3846 (79.5)		63445 (94.8)	20941 (93.8)	5766 (89.1)	2368 (88.5)	
Oily fish intake, *n* (%)					< 0.001					< 0.001
Never	4807 (6.7)	1872 (8.4)	1149 (9.7)	598 (12.4)		5129 (7.7)	2041 (9.1)	819 (12.6)	317 (11.8)	
Less than once a week	18999 (26.6)	6341 (28.5)	3266 (27.6)	1322 (27.3)		20315 (30.3)	7129 (31.9)	1965 (30.3)	838 (31.3)	
Once a week	30849 (43.1)	9102 (40.9)	4925 (41.6)	1918 (39.7)		27040 (40.4)	8666 (38.8)	2399 (37.1)	979 (36.6)	
More than twice a week	16877 (23.6)	4926 (22.1)	2490 (21.0)	999 (20.7)		14464 (21.6)	4492 (20.1)	1292 (20.0)	542 (20.3)	
Poultry intake, *n* (%)					< 0.001					< 0.001
Never	3469 (4.8)	702 (3.2)	680 (5.7)	193 (4.0)		2192 (3.3)	486 (2.2)	350 (5.4)	84 (3.1)	
Less than once a week	8775 (12.3)	2217 (10.0)	1426 (12.1)	508 (10.5)		8892 (13.3)	2732 (12.2)	997 (15.4)	389 (14.5)	
Once a week	28342 (39.6)	7994 (35.9)	4682 (39.6)	1781 (36.8)		27909 (41.7)	8793 (39.4)	2668 (41.2)	1057 (39.5)	
More than twice a week	30946 (43.3)	11328 (50.9)	5042 (42.6)	2355 (48.7)		27955 (41.8)	10317 (46.2)	2460 (38.0)	1146 (42.8)	
Processed meat intake, *n* (%)					< 0.001					< 0.001
Never	9044 (12.6)	1866 (8.4)	1564 (13.2)	468 (9.7)		3455 (5.2)	612 (2.7)	500 (7.7)	116 (4.3)	
Less than once a week	29196 (40.8)	7975 (35.9)	4482 (37.9)	1644 (34.0)		16120 (24.1)	4456 (20.0)	1372 (21.2)	526 (19.7)	
Once a week	20202 (28.2)	6897 (31.0)	3484 (29.5)	1509 (31.2)		20882 (31.2)	6859 (30.7)	1943 (30.0)	760 (28.4)	
More than twice a week	13090 (18.3)	5503 (24.7)	2300 (19.4)	1216 (25.1)		26491 (39.6)	10401 (46.6)	2660 (41.1)	1274 (47.6)	
Beef intake, *n* (%)					< 0.001					< 0.001
Never	8662 (12.1)	1947 (8.8)	1630 (13.8)	572 (11.8)		4743 (7.1)	935 (4.2)	734 (11.3)	207 (7.7)	
Less than once a week	33892 (47.4)	10151 (45.6)	5565 (47.0)	2192 (45.3)		30735 (45.9)	9000 (40.3)	2837 (43.8)	1021 (38.2)	
Once a week	21203 (29.6)	7272 (32.7)	3458 (29.2)	1551 (32.1)		23278 (34.8)	8723 (39.1)	2126 (32.8)	989 (37.0)	
More than twice a week	7775 (10.9)	2871 (12.9)	1177 (9.9)	522 (10.8)		8192 (12.2)	3670 (16.4)	778 (12.0)	459 (17.2)	
Pork intake, *n* (%)					< 0.001					< 0.001
Never	12729 (17.8)	3286 (14.8)	2500 (21.1)	853 (17.6)		8249 (12.3)	2173 (9.7)	1177 (18.2)	349 (13.0)	
Less than once a week	41292 (57.7)	12395 (55.7)	6275 (53.0)	2507 (51.8)		39443 (58.9)	12261 (54.9)	3351 (51.8)	1317 (49.2)	
Once a week	15813 (22.1)	5835 (26.2)	2743 (23.2)	1307 (27.0)		16828 (25.1)	6699 (30.0)	1628 (25.1)	837 (31.3)	
More than twice a week	1698 (2.4)	725 (3.3)	312 (2.6)	170 (3.5)		2428 (3.6)	1195 (5.4)	319 (4.9)	173 (6.5)	
Lamb intake, *n* (%)					< 0.001					< 0.001
Never	12630 (17.7)	3451 (15.5)	2263 (19.1)	814 (16.8)		8668 (12.9)	2528 (11.3)	1066 (16.5)	397 (14.8)	
Less than once a week	40431 (56.5)	12284 (55.2)	6343 (53.6)	2552 (52.8)		37814 (56.5)	11904 (53.3)	3337 (51.5)	1294 (48.4)	
Once a week	16736 (23.4)	5821 (26.2)	2896 (24.5)	1296 (26.8)		18166 (27.1)	6888 (30.8)	1743 (26.9)	835 (31.2)	
More than twice a week	1735 (2.4)	685 (3.1)	328 (2.8)	175 (3.6)		2300 (3.4)	1008 (4.5)	329 (5.1)	150 (5.6)	
Milk intake, *n* (%)					< 0.001					< 0.001
No	2196 (3.1)	745 (3.3)	443 (3.7)	196 (4.1)		1909 (2.9)	710 (3.2)	241 (3.7)	98 (3.7)	
Yes	69336 (96.9)	21496 (96.7)	11387 (96.3)	4641 (95.9)		65039 (97.1)	21618 (96.8)	6234 (96.3)	2578 (96.3)	
Coffee intake, mean (SD), cups	1.97 (1.81)	2.11 (1.98)	1.85 (1.86)	1.99 (2.15)	< 0.001	2.11 (1.97)	2.24 (2.13)	1.95 (2.05)	2.06 (2.28)	< 0.001
Fresh fruit intake, *n* (%)					< 0.001					< 0.001
0–1 pieces/day	17206 (24.1)	5726 (25.7)	3222 (27.2)	1289 (26.6)		27013 (40.3)	8729 (39.1)	2817 (43.5)	1052 (39.3)	
2–3 pieces/day	39561 (55.3)	11955 (53.8)	6230 (52.7)	2515 (52.0)		30876 (46.1)	9879 (44.2)	2794 (43.2)	1135 (42.4)	
≥ 3 pieces/day	14765 (20.6)	4560 (20.5)	2378 (20.1)	1033 (21.4)		9059 (13.5)	3720 (16.7)	864 (13.3)	489 (18.3)	
Vegetable intake, mean (SD), tablespoons/day	5.23 (3.05)	5.19 (3.05)	5.12 (3.24)	5.18 (3.35)	0.005	4.92 (3.28)	4.98 (3.31)	4.85 (4.04)	4.98 (3.72)	0.005
Cardiovascular disease, *n* (%)					< 0.001					< 0.001
No	64815 (90.6)	3758 (16.9)	10130 (85.6)	3610 (74.6)		53363 (79.7)	15321 (68.6)	4653 (71.9)	1574 (58.8)	
Yes	6717 (9.4)	18483 (83.1)	1700 (14.4)	1227 (25.4)		13585 (20.3)	7007 (31.4)	1822 (28.1)	1102 (41.2)	
Cerebrovascular disease, *n* (%)					< 0.001					< 0.001
No	67009 (93.7)	20436 (91.9)	10671 (90.2)	4247 (87.8)		60282 (90.0)	19327 (86.6)	5385 (83.2)	2124 (79.4)	
Yes	4523 (6.3)	1805 (8.1)	1159 (9.8)	590 (12.2)		6666 (10.0)	3001 (13.4)	1090 (16.8)	552 (20.6)	
Diabetes, *n* (%)					< 0.001					< 0.001
No	67709 (94.7)	17872 (80.4)	10838 (91.6)	3555 (73.5)		59952 (89.6)	15824 (70.9)	5280 (81.5)	1552 (58.0)	
Yes	3823 (5.3)	4369 (19.6)	992 (8.4)	1282 (26.5)		6996 (10.4)	6504 (29.1)	1195 (18.5)	1124 (42.0)	
Polygenic risk score, *n* (%)					0.154					0.084
Low	34863 (48.7)	10971 (49.3)	5689 (48.1)	2342 (48.4)		32482 (48.5)	11034 (49.4)	3135 (48.4)	1278 (47.8)	
High	36669 (51.3)	11270 (50.7)	6141 (51.9)	2495 (51.6)		34466 (51.5)	11294 (50.6)	3340 (51.6)	1398 (52.2)	

### Associations of obesity, sarcopenia, and sarcopenic obesity with incident dementia risk

**Fig. 2:**
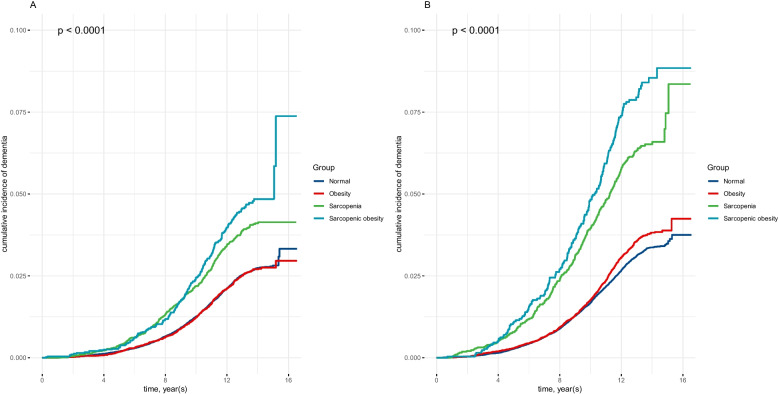
Kaplan-Meier curves of associations between obesity, sarcopenia, and sarcopenic obesity and dementia incident for women (**A**) and men (**B**)

After a median follow-up of 13.42 years, there were 2941 and 3231 incident dementia cases respectively for women and men. We constructed Kaplan-Meier curves (Fig. 2) to compare cumulative incidence of all-cause dementia across different groups. The results showed that for both women and men, those with sarcopenic obesity had the highest incidence of dementia, and those with sarcopenia came to the second. However, no difference of cumulative incidence of dementia was found between normal and obesity group.

We used time-varying Cox proportional hazards models to examine the associations of different groups with the dementia risk (Table 2). In the unadjusted and age-adjusted models, compared to participants in normal group, those with sarcopenia or sarcopenic obesity were associated with an elevated dementia risk. In the multivariable adjusted models, associations of sarcopenia (women: HR = 1.719 [1.429–2.068] (< 8 years) and 1.156 [1.015–1.317] (≥ 8 years); men: HR = 2.158 [1.792–2.599] (< 8 years) and 1.440 [1.244–1.665] (≥ 8 years)) or sarcopenic obesity (women: HR = 1.424 [1.227–1.653]; men: HR = 1.989 [1.702–2.323]) with dementia risk remained significant. However, we observed no association of obesity with incident dementia risk among women in all models. Among men, significant association was observed in unadjusted model and age-adjusted model, but not in multivariable model. In multivariable model, AR% of sarcopenia was 41.827% (< 8 years) and 13.494% (≥ 8 years) for women; it was 53.661% (< 8 years) and 30.556% (≥ 8 years) for men. AR% of sarcopenic obesity was 29.775% and 49.723% for women and men, respectively (Table 2).

**Table 2:** Associations of obesity, sarcopenia, and sarcopenic obesity with all-cause dementia incident

	Women		Men	
	HR (95%CI)	AR%	HR (95%CI)	AR%
Unadjusted model				
Normal	Ref	-	Ref	-
Obesity	0.993 (0.902–1.094)	NA	1.130 (1.037–1.230)	11.504%
Sarcopenia ^#^	2.030 (1.689–2.440)	50.739%	2.695 (2.243–3.240)	62.894%
Sarcopenia ^§^	1.347 (1.184–1.533)	25.760%	1.766 (1.530–2.039)	43.375%
Sarcopenic obesity	1.824 (1.578–2.107)	45.175%	2.649 (2.275–3.083)	62.250%
Age-adjusted model				
Normal	Ref	-	Ref	-
Obesity	0.999 (0.907–1.100)	NA	1.160 (1.065–1.263)	13.793%
Sarcopenia ^#^	1.807 (1.503–2.172)	44.660%	2.380 (1.980–2.861)	57.983%
Sarcopenia ^§^	1.205 (1.058–1.371)	20.747%	1.564 (1.355–1.806)	36.061%
Sarcopenic obesity	1.676 (1.451–1.937)	40.334%	2.436 (2.092–2.836)	58.949%
Multivariable model				
Normal	Ref	-	Ref	-
Obesity	0.920 (0.833–1.016)	NA	1.044 (0.957–1.140)	NA
Sarcopenia ^#^	1.719 (1.429–2.068)	41.827%	2.158 (1.792–2.599)	53.661%
Sarcopenia ^§^	1.156 (1.015–1.317)	13.494%	1.440 (1.244–1.665)	30.556%
Sarcopenic obesity	1.424 (1.227–1.653)	29.775%	1.989 (1.702–2.323)	49.723%

Multivariable model was adjusted by baseline age, Townsend Deprivation Index (TDI), ethnicity (White, Asian or Asian British, Black or Black British, and other), education qualifications (degree or no degree), physical activity (low, moderate and high level), smoking status (current, former, or never users), alcohol status (current, former, or never users), and vegetables consumption (continuous), fruits consumption (0–1, 2–3, and ≥ 3 pieces per day), red meat consumption (never, less than once a week, once a week, and more than twice a week), processed meat consumption (never, less than once a week, once a week, and more than twice a week), and oily fish consumption (never, less than once a week, once a week, and more than twice a week), coffee (continuous) and dairy (yes or no). Normal group consisted of those without sarcopenia, obesity, or sarcopenic obesity. *HR*, hazard ratio; *AR%*, attributable risk proportion; *NA*, not applicable

^#^Effect of sarcopenia on all-cause incident dementia risk while duration is less than 8 years

^§^Effect of sarcopenia on all-cause incident dementia risk while duration is no less than 8 years

### Subgroup analysis and sensitivity analysis

**Fig. 3:**
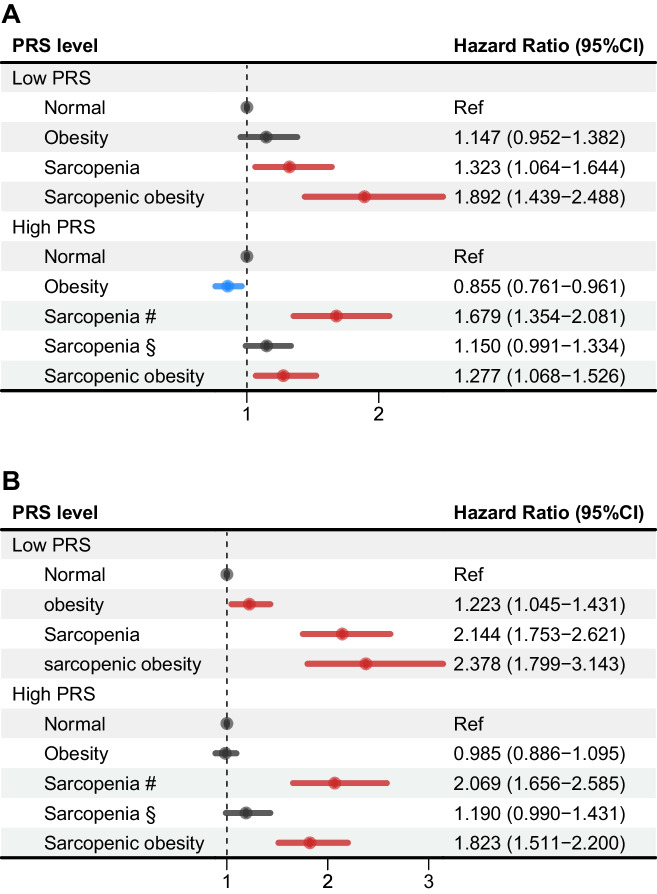
Associations of obesity, sarcopenia, and sarcopenic obesity with incident dementia stratified by PRS level for women (**A**) and men (**B**). Stratified analysis was based on multivariable model, which was adjusted by baseline age, Townsend Deprivation Index (TDI), ethnicity (White, Asian or Asian British, Black or Black British, and other), education qualifications (degree or no degree), physical activity (low, moderate and high level), smoking status (current, former, or never), alcohol status (current, former, or never), vegetables consumption, fruits (0–1, 2–3, and ≥ 3 pieces per day), red meat consumption (never, less than once a week, once a week, and more than twice a week), processed meat consumption (never, less than once a week, once a week, and more than twice a week), and oily fish consumption (never, less than once a week, once a week, and more than twice a week), coffee, and dairy (yes or no). Normal group consisted of those without sarcopenia, obesity, or sarcopenic obesity. *P* value for interaction between three different conditions and PRS level were 0.0480 and < 0.001, respectively for women and men

We conducted stratified analyses by PRS level based on multivariable adjusted model (Fig. 3). In low PRS level, association between sarcopenia and dementia risk remained similar (HR_women_ = 1.323 [1.064–1.644]; HR_men_ = 2.144 [1.753–2.621]). In high PRS level, however, significant associations were only observed when follow-up time was less than 8 years (HR_women_ = 1.679 [1.355–2.081]; HR_men_ = 2.069 [1.656–2.585]). Additionally, the results of association between sarcopenic obesity and dementia risk remained similar for both women (HR_low-PRS_ = 1.892 [1.439–2.488], and HR_high-PRS_ = 1.277 [1.068–1.526]) and men (HR_low-PRS_ = 2.378 (1.799–3.143) and HR_high-PRS_ = 1.823 (1.511–2.200)). When compared to participants in normal group, we still observed no association of obesity with incident dementia among low PRS level women, but we did find a significant inverse association among high PRS level women (HR = 0.855 [0.761–0.961]). Additionally, obesity was associated with a higher risk for dementia among low PRS level men (HR = 1.223 [1.045–1.431]), but no significant association was observed among high PRS level men. Moreover, we also conducted two sensitivity analyses, and results showed no substantial change when we excluded participants who developed dementia during the first year of follow-up or considered the competing risk of death (Additional file 1: Table S1).

### Associations of obesity, sarcopenia, and sarcopenic obesity with dementia onset

We conducted RMST models to investigate the associations of obesity, sarcopenia and sarcopenic obesity with dementia onset (Table 3). In comparison to women in normal group, we observed that women with obesity were diagnosed as dementia with 1.114 (1.100, 1.128) years delayed, while those with sarcopenia and sarcopenic obesity were respectively diagnosed with 0.080 (0.078, 0.081) years and 0.109 (0.092, 0.126) years in advance.

**Table 3:** Associations of obesity, sarcopenia, and sarcopenic obesity with onset of incident dementia for women and men

	Women		Men	
	RMST differences (95% CI), year(s)	RMST ratio (95% CI)	RMST differences (95% CI), year(s)	RMST ratio (95%CI)
Normal	Ref	Ref	Ref	Ref
Obesity	1.114 (1.100, 1.128)	1.075 (1.071, 1.079)	− 0.170 (− 0.190, − 0.151)	0.989 (0.988, 0.990)
Sarcopenia	− 0.080 (− 0.081, − 0.078)	0.995 (0.995, 0.995)	− 0.192 (− 0.195, − 0.189)	0.987 (0.987, 0.987)
Sarcopenic obesity	− 0.109 (− 0.126, − 0.092)	0.993 (0.992, 0.994)	− 0.511 (− 0.535, − 0.487)	0.968 (0.966, 0.969)

*RMST*, restricted mean survival time; RMST model was adjusted by baseline age, Townsend Deprivation Index (TDI), ethnicity (White, Asian or Asian British, Black or Black British, and other), education qualifications (degree or no degree), physical activity (low, moderate and high level), smoking status (current, former, or never), alcohol status (current, former, or never), vegetables consumption, fruits consumption (0–1, 2–3, and ≥ 3 pieces per day), red meat consumption (never, less than once a week, once a week, and more than twice a week), processed meat consumption (never, less than once a week, once a week, and more than twice a week), and oily fish consumption (never, less than once a week, once a week, and more than twice a week), coffee (continuous), and dairy (yes or no). Normal group consisted of those without sarcopenia, obesity, or sarcopenic obesity

However, we found different results among men. In comparison to men in normal group, obesity (0.170 (0.151, 0.190) years), sarcopenia (0.192 (0.189, 0.195) years), and sarcopenic obesity (0.511 (0.487, 0.535) years) will all advance the onset of incident dementia to varying degree.

### Associations of obesity, sarcopenia, and sarcopenic obesity with brain structure

In secondary analysis, we employed linear regression models to explore the associations of obesity, sarcopenia, and sarcopenic obesity with brain structure (Table 4). Compared to the normal group, obese women related to larger volume of total brain and white matter. However, different results were found in men. The volume of total brain, grey matter, and WMHs were associated with obesity in men. We noticed that sarcopenia was associated with lower grey matter in hippocampus for both women and men. Moreover, men with sarcopenic obesity had a lower grey matter volume and a higher WMHs volume than normal participants.

**Table 4:** Associations of obesity, sarcopenia, and sarcopenic obesity with brain structure for women and men

	***β*** coefficients and 95% CIs in volume of different brain regions				
	Total brain *	White matter *	Grey matter *	Grey matter in hippocampus	White matter hyperintensities^#^
Women (*n* = 6328)					
Normal	Ref	Ref	Ref	Ref	Ref
Obesity	6751 (2549, 10953)	8238 (5362, 11114)	− 1486 (− 4026, 1053)	− 39 (− 90, 13)	0.05 (− 0.02, 0.11)
Sarcopenia	− 3229 (− 8677, 2220)	− 1849 (− 5568, 1871)	− 1380 (− 4693, 1933)	− 145 (− 212, − 78)	0.06 (− 0.02, 0.14)
Sarcopenic obesity	3429 (− 8426, 15284)	6675 (− 1440, 14789)	− 3246 (− 10412, 3919)	− 108 (− 254, 38)	0.16 (− 0.02, 0.34)
Men (*n* = 7348)					
Normal	Ref	Ref	Ref	Ref	Ref
Obesity	− 12677 (− 16464, − 8891)	2172 (− 428, 4771)	− 14850 (− 17170, − 12528)	− 21 (− 71, 28)	0.25 (0.19, 0.31)
Sarcopenia	− 2536 (− 9963, 4892)	1696 (− 3412, 6804)	− 4232 (− 8779, 316)	− 108 (− 205, − 12)	0.05 (− 0.06, 0.17)
Sarcopenic obesity	− 11221 (− 25357, 2915)	3423 (− 6281, 13127)	− 14640 (− 23309, − 5978)	− 174 (− 358, 10)	0.26 (0.05, 0.48)

*The volume of total brain, white matter, and grey matter were all normalized for the external surface of the skull

^#^The volume of white matter hyperintensities was log-transformed

### Mediation analysis

**Fig. 4:**
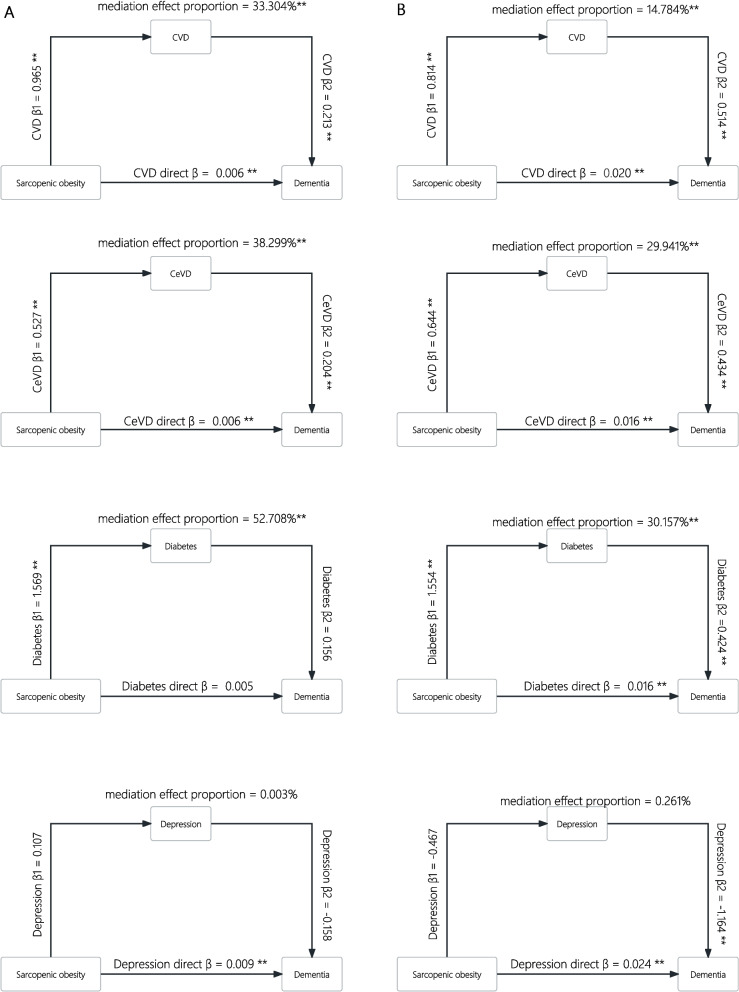
Mediations effect caused by CVD, CeVD, and diabetes of the association between sarcopenic obesity and incident dementia in women (**A**) and men (**B**)

In the association between sarcopenic obesity and incident dementia, we examined CVD, CeVD, diabetes and depression as potential mediators (Fig. 4). Results showed that 33.304%, 38.299%, and 52.708% of total association between sarcopenic obesity and incident dementia was respectively mediated by CVD, CeVD, and diabetes for women and 14.784%, 29.941%, and 30.157% for men. No significant mediation effect of depression was observed.

## Discussion

In this population-based study, we investigated the associations of obesity, sarcopenia, and sarcopenic obesity with dementia risk stratified by sex and further explored the roles of genetic background and chronic diseases. Importantly, we also examined the associations of these three different conditions with dementia onset and brain structure. Our findings implicate sarcopenic obesity or sarcopenia have a greater contribution to dementia and significantly advanced dementia onset than singular obesity.

### Associations of obesity, sarcopenia, and sarcopenic obesity with incident dementia risk

Our findings are novel and differ from previous studies on the association between obesity and dementia. For example, Guo et al. found that obesity was negatively associated with cognitive level in elderly women, while obese men exhibited better cognitive level [37]. Hu et al. detected positive association of obesity with cognitive function in older women, but no significant association was observed among older men [38]. However, reverse causality may exist in these two studies due to the cross-sectional design. A prospective study conducted among Finland women reported that participants with high BMI had a lower dementia risk; however, low identification rates of dementia may lead to the misclassification bias [39]. Another prospective study indicated that obesity was associated with increased long-term dementia risk; yet important covariates such as physical activity and dietary intake were not adjusted, contributing to an unneglectable confounding effect [7]. However, findings from several recent large cohort studies suggested that obesity is not an independent risk factor to dementia, which is also supported by our primary analysis [40, 41]. On the other hand, our finding that sarcopenia is associated with elevated dementia risk was in line with previous studies. For instance, Lin et al. observed that sarcopenia was significantly associated with lower cognitive function and higher Rotterdam Study Basic Dementia Risk Model score [8]. Beeri et al. noticed that baseline sarcopenia was associated with a higher risk of incident AD, within 5.6 years average follow-up period [42]. Importantly, our study added the evidence that sarcopenic obesity or sarcopenia contributed to an elevated dementia risk, which was larger than singular obesity brought. This significance was also observed in subsequent PRS stratified analyses, which suggested the sarcopenia obesity might be served as an explanation of “obesity paradox.”

The following mechanisms may help understand. Obesity contributes to a really complicated alteration on the homeostasis of adipokines, affecting dementia risk with an unknown direction. For example, obesity is associated with secretion of nerve growth factor, who has been proposed as a protector against dementia via the cholinergic system [43]. Conversely, obesity is linked to surged dose of interleukin 6, who presents a negative impact on synaptic plasticity and neurogenesis [43]. Hence, the direction of pooling effect from obesity is undetermined. However, apart from ambiguous effect by obesity on dementia, sarcopenia is characterized with the disorder of myokines, which eventuates evident harms [44]. It has been demonstrated that myokines exert a great influence on neurological function by regulating microglial polarization, activating astrocyte, modulating signaling of insulin and neuroinflammation in neurons, and altering emotional and cognitive processing [45], among which irisin, who is reported to be associated with sarcopenia, plays vital role [46]. The secretion of irisin involves an interactive process, whereby its release promotes muscle biogenesis while being concurrently boosted by muscle growth [47]. Additionally, previous studies indicated that irisin may exist neuroprotection probably due to enhancing BDNF concentration in the hippocampus [48]. Consequently, sarcopenic obesity or sarcopenia may pose a greater risk for developing dementia than singular obesity. Notably, some research has implicated that interaction of adipokines and myokines also contributes to a more profound pro-inflammatory milieu than their singular existence [44]. Additionally, precious study showed obesity also relates to low level of serum irisin, which suggested that the coexistence of obesity and sarcopenia may further diminish the neuroprotective effect [49]. Due to limitation of statistical methods, however, we did not find sufficient evidence to support this assertion, which requires for more studies to be demonstrated. In our studied population, AR% of sarcopenic obesity on dementia was 29.775% and 49.723% respectively for women and men, indicating existence of sarcopenic obesity may contribute to a large number of dementia cases. Given the such high prevalence and enormous perniciousness of sarcopenic obesity, compared to the measures of weight loss, increasing muscle mass and strength to prevent sarcopenic obesity may obtain more benefits for cognitive fitness.

Interestingly, our further analyses stratified by PRS level suggested that obese women with high PRS level had lower dementia risk. We supposed that estrogen may account for such result. The European Prevention of Alzheimer’s Disease cohort study implicated that estrogen therapy could improve cognition at-risk APOE4 women [50]. It is assumed that although the women with APOE4 are more susceptible to dementia, they may obtain greater benefits from estrogen therapy. Additionally, obesity has been shown to be related to increased estrogen level, so it is plausible that women with APOE4 may derive extra benefits from obesity [51]. In contrast to that, our studies observed obese men with low PRS level had higher dementia risk, whose reasons behind are yet unclear. Therefore, the difference and interaction of sex and genes remain to be excavated. On the other hand, stratified analyses suggested that the association between sarcopenia and dementia risk disappeared when follow-up time is no less than 8 years. We hypothesized that late-life sarcopenia may have little impact to dementia risk in those who with high PRS, in which situation genetic factors may play the key role in dementia developing.

### Associations of obesity, sarcopenia, and sarcopenic obesity with incident dementia onset

To further understand the roles of obesity, sarcopenia, and sarcopenic obesity in dementia developing, to the best of our knowledge, this study represents the first exploration of their associations with the onset of dementia. Interestingly, distinct sex-specific associations of obesity with dementia onset were found. In women, obesity was significantly associated with a delayed onset of dementia. As mentioned above, obesity relates to increased estrogen, who modulates synaptic plasticity and brain derived neurotrophic factor expression in the hippocampus, contributing to neuroprotection, and such effect was only observed in female, but not in male [52]. For men, our analysis showed that obesity was related to a significant early onset of dementia. Obesity contributes to decreased testosterone [53], who also presents protective effect on dementia developing, by increasing cleavage of the β-amyloid precursor protein to enhance secretion of non-amyloidogenic fragments in hypothalamus and preventing β-amyloid-induced cell death in hippocampus [54, 55]. Animal studies indicated that testosterone can dose-dependently increase neurogenesis with long exposures in males but not in females [52]. Hence, we hypothesized the associations of obesity with dementia onset differ among women and men due to sex hormone mechanisms.

Additionally, we observed sarcopenia is associated with advanced dementia onset (women: 0.08 years; men: 0.192 years). Of note, we detected that sarcopenic obesity significantly advanced dementia onset, by approximately 0.109 years in women and 0.511 years in men. Therefore, there is a public health significance of preventing sarcopenia and sarcopenic obesity, which could delay onset of dementia and contribute to reduce the disease burden for individuals, families, and society.

### Associations of obesity, sarcopenia, and sarcopenic obesity with brain structure

We put forward the previous studies by illustrating that the roles of obesity, sarcopenia, and sarcopenic obesity in dementia developing may account to their impacts on certain brain regions and these impacts may vary by sex. Previous studies indicated that dementia was associated with grey matter deterioration [56], white matter degeneration [57], and WMHs accumulation [58]. Our results found that obese men were associated with grey matter atrophy and larger WMHs, which aligns to previous studies [13, 14]. Interestingly, we found obese women had a larger volume of white matter, while a study by Ronan et al. observed an opposite result, however, whose reliability is limited by small sample size [59]. Given such sex-specific association, we posed the assumption that obesity may benefit women against dementia by protecting brain white matter but lead to degeneration of brain grey matter, accelerating the dementia onset in men. Sarcopenia was found to be associated with smaller grey matter in hippocampus, which may be the mechanism how it prompts development of dementia. Sarcopenic obesity may contribute to dementia by lowing grey matter volume and increasing WMHs. More studies should be conducted to clarify and explain the above findings.

### Mediations effect on association between sarcopenic obesity and dementia

With mediation analyses, our analyses revealed significant mediation effects of CVD, CeVD, and diabetes, supported by a growing body of evidence. One systematic reviews encompassing 12 researches indicated that sarcopenic obesity is prone to increase CVD risk in older people [15]. And a study among Mongolian adults showed that sarcopenic obesity was associated with higher stroke incidence [17]. Besides, a meta-analysis has reported that sarcopenic obesity was related to increased diabetes risk [19]. On the other hand, CVD, CeVD, and diabetes all have been considered as risk factors for dementia [16, 18, 20], which might be related to the accumulation of abnormally folded amyloid-β peptides and tau proteins [60], and vascular damage, especially the small vessel arteriosclerosis [61]. Hence, apart from the direct harmful effect, sarcopenic obesity may also elevate the risk of chronic diseases, consequently leading to the dementia events. However, no significant mediated effect of depression was found.

### Limitations and strengths

In this study, we investigated the associations of obesity, sarcopenia, and sarcopenic obesity with dementia risk in a large prospective cohort, comprising almost half a million participants, which provided adequate statistical power and a large number of dementia events. Moreover, to the best of our knowledge, it is the first study to explore and compare the associations of obesity, sarcopenia, and sarcopenic obesity with dementia onset, whose results yielded a public health significance. Furthermore, in-depth information was available on socioeconomic characteristics, genetic factors, lifestyle habits, chronic diseases, and other covariates for minimizing confounding effect and testing the robustness of the results.

We acknowledge limitations. Firstly, the majority of participants in our cohort were of British “white” ethnicity, and further studies are required among diverse ethnic populations to enhance the generalizability. Secondly, mild dementia may go undetected leading to misclassification bias; nevertheless, positive predictive value of the defined dementia outcome in UK Biobank has been identified to be acceptable in previous study [62]. Thirdly, our study employed the BIA to estimate muscle quality, while MRI and computed tomography are considered as the gold standards for non-invasive assessment of muscle quantity; yet EWGSOP2 indicated that BIA-based measurements is also ideal approach to estimate muscle quantity, especially in a large-scale study [27]. Fourthly, despite our efforts to consider several potential confounders, unknown confounding effects may still present in our study. Fifth, given the variations among different ethnic groups, it may not be rigorous to directly use BMI ≥ 30 kg/m^2^ to define obesity. Finally, the mediators between sarcopenic obesity and dementia were not fully examined. Based on previous evidence, we only included CVD, CeVD, diabetes, and depression for mediation analysis, but other chronic conditions may also affect the association which requires for further exploration.

## Conclusions

Our study offers novel insights into the relationship of obesity, sarcopenia, and particularly sarcopenic obesity with dementia risk. Sarcopenic obesity emerged as a significant contributor to increased dementia risk and notably accelerated dementia onset in both men and women. Additionally, sarcopenia was associated with higher dementia risk. Intriguingly, the role of obesity in the development of dementia may vary by sex and genetic susceptibility. One of potential mechanisms might be related to the different roles of obesity, sarcopenia, and sarcopenic obesity in brain structure variations. Our study challenges the traditional focus on weight loss as a sole preventive measure for dementia and emphasizes the critical importance of enhancing and maintaining muscle mass and strength through a balanced diet and adequate resistance training in the fight against dementia.

### Supplementary Information

**Additional file 1:.** Figure S1 directed acyclic graph showing potential confounders and mediators; Table S1 Sensitivity analyses

## Data Availability

Our access to data from the UK Biobank cohort was approved by the UKB Ethics Advisory Committee (Application ID: 91486). All researchers will be subject to the same application process and approval criteria as specified by UK Biobank.

## Abbreviations

HRs: Hazard ratios
CIs: Confidence intervals
CVD: Cardiovascular disease
CeVD: Cerebrovascular disease
PRS: Polygenic risk score
UKB: UK Biobank
APOE4: Apolipoprotein E epsilon 4
EWGSOP2: European Working Group on Sarcopenia in Older People 2
SMI: Skeletal muscle mass index
BIA: Bioelectrical impedance analysis
ASM: Appendicular skeletal muscle mass
HES: Hospital Episode Statistics for England
SMR: Scottish Morbidity Record
PEDW: Patient Episode Database for Wales
NHS: National Health Service
ICD-10: International Classification of Diseases 10th revision
MRI: Magnetic resonance imaging
WMHs: White matter hyperintensities
TDI: Townsend Deprivation Index
AD: Alzheimer’s disease
IPAQ: International Physical Activity Questionnaire
MICE: Multiple imputation chain-equation
AR%: Attributable risk proportion
RMST: Restricted mean survival time
DAG: Directed acyclic graph
