# Supplementary material for: Sarcopenic obesity is part of obesity paradox in dementia development: evidence from a population-based cohort study
Source: BMC Med. 2024 Mar 22;22:133. doi: 10.1186/s12916-024-03357-4 (PMC10960494; doi:10.1186/s12916-024-03357-4)
Supplement: Supplementary file 1 — Additional file 1:. Figure S1 directed acyclic graph showing potential confounders and mediators; Table S1 Sensitivity analyses [file 12916_2024_3357_MOESM1_ESM.docx]

Additional file 1


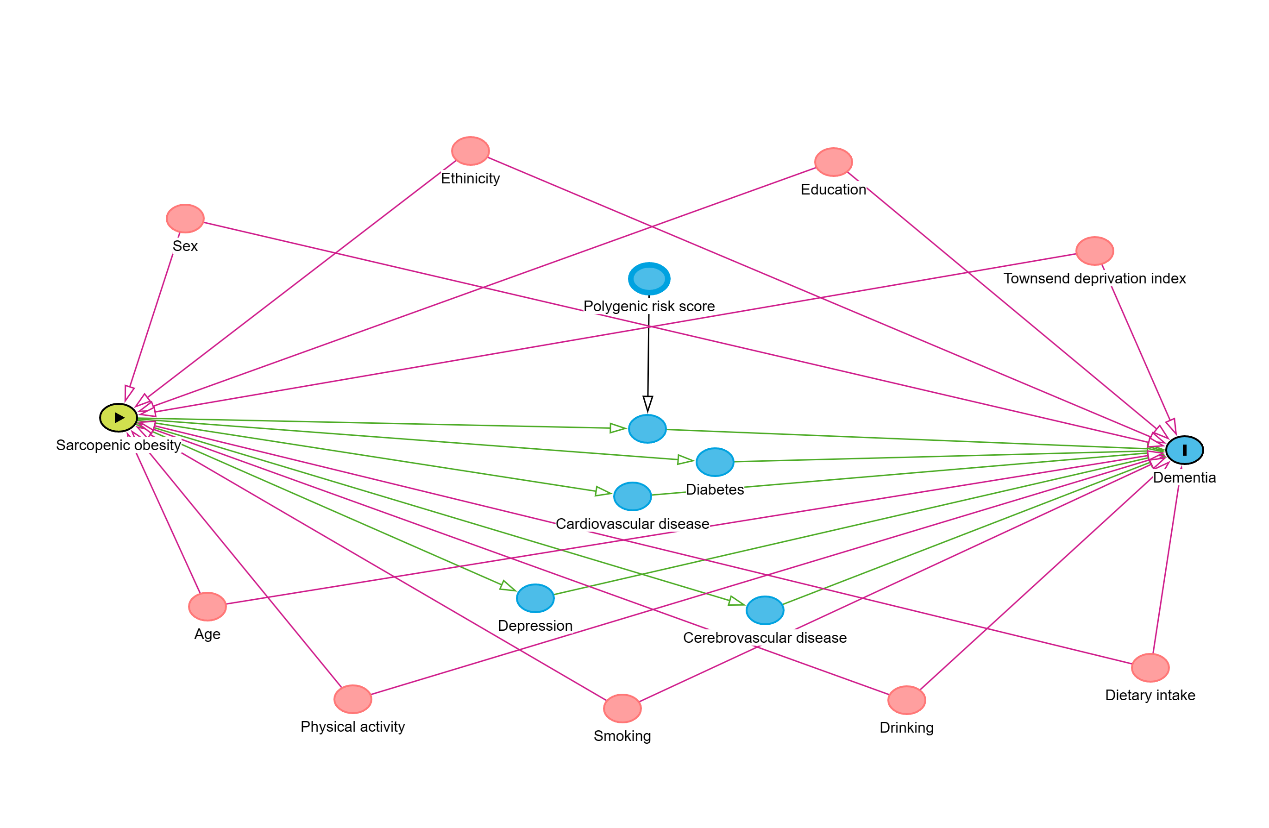


**Figure S1 directed acyclic graph showing potential confounders and mediators**

**Table S1 Sensitivity analyses**

|  | Women |  | Men |
| --- | --- | --- | --- |
|  | HR (95%CI) |  | HR (95%CI) |
| Sensitivity model1# |  |  |  |
| Normal | Ref |  | Ref |
| Obesity | 0.918 (0.832, 1.014) |  | 1.049 (0.961, 1.145) |
| Sarcopenia ^#^ | 1.731 (1.438, 2.823) |  | 2.192 (1.819, 2.643) |
| Sarcopenia ^§^ | 1.156 (1.015, 1.317) |  | 1.439 (1.244, 1.665) |
| Sarcopenic obesity | 1.413 (1.216, 1.641) |  | 1.994 (1.706, 2.331) |
| Sensitivity model2* |  |  |  |
| Normal | Ref |  | Ref |
| Obesity | 0.922 (0.835, 1.017) |  | 1.029 (0.943, 1.122) |
| Sarcopenia | 1.275 (1.146, 1.419) |  | 1.577 (1.405, 1.771) |
| Sarcopenic obesity | 1.412 (1.216, 1.639) |  | 1.876 (1.602, 2.197) |

#: Sensitivity model1 is based on multivariable model, but excluded participants who had a dementia incident during the first year.

*: Sensitivity model2 is established to assess the influence of death as a competing risk.

Both sensitivity model1 and 2 was adjusted by baseline age, Townsend Deprivation Index (TDI), ethnicity (White, Asian or Asian British, Black or Black British, and other), education qualifications (degree or no degree), physical activity (low, moderate and high level), smoking status (current, former, or never), alcohol status (current, former, or never), vegetables consumption, fruits (0-1, 2-3, and ≥ 3 pieces per day), red meat consumption (never, less than once a week, once a week, and more than twice a week), processed meat consumption (never, less than once a week, once a week, and more than twice a week), and oily fish consumption (never, less than once a week, once a week, and more than twice a week), coffee, and dairy (yes or no). Normal group consisted of those without sarcopenia, obesity, or sarcopenic obesity.
